# A novel multi-variate immunological approach, reveals immune variation associated with environmental conditions, and co-infection in the koala (*Phascolarctos cinereus*)

**DOI:** 10.1038/s41598-024-57792-7

**Published:** 2024-03-27

**Authors:** Cristina M. Fernandez, Mark B. Krockenberger, Valentina, S. A. Mella, Belinda R. Wright, Mathew S. Crowther, Damien P. Higgins

**Affiliations:** 1 Faculty of Science, Sydney School of Veterinary Science, The University of Sydney, Sydney, NSW 2006 Australia; 2 Sydney Infectious Diseases, The University of Sydney, 176 Hawkesbury Road, Westmead, NSW 2145 Australia; 3 School of Life and Environmental Sciences, The University of Sydney, Sydney, NSW 2006 Australia

**Keywords:** Animal physiology, Immunology, Applied immunology, Cytokines, Infectious diseases

## Abstract

External signs of disease are frequently used as indicators of disease susceptibility. However, immune profiling can be a more effective indicator to understand how host responses to infection may be shaped by host, pathogen and environmental factors. To better inform wildlife health assessment and research directions, we investigated the utility of a novel multivariate immunophenotyping approach examining innate and adaptive immune responses in differing climatic, pathogen co-infection and demographic contexts across two koala (*Phascolarctos cinereus)* populations in New South Wales: the Liverpool Plains (LP), and Southern Highlands to South-west Sydney (SHSWS). Relative to the comparatively healthy SHSWS, the LP had greater and more variable innate immune gene expression (IL-1β, IL-6), and KoRV transcription. During extreme heat and drought, koalas from the LP displayed upregulation of a stress pathway gene and reduced adaptive immune genes expression, haematocrit and plasma protein, suggesting the possibility of environmental impacts through multiple pathways. In those koalas, KoRV transcription status, *Chlamydia pecorum* infection loads, and visible urogenital inflammation were not associated with immune variation, suggesting that immune markers were more sensitive indicators of real-time impacts than observed disease outcomes.

## Introduction

Clinical signs are often used as an outcome variable when investigating the impacts of host, pathogen or environmental factors on disease susceptibility. This is problematic when studying drivers of wildlife disease as external signs of disease alone may not reflect internal pathological processes^1,2^ and only animals exposed to infection can be considered challenged when evaluating host susceptibility. In cross sectional studies it can be impossible to determine if clinical signs reflect current factors or those that induced a pathway of chronic disease months to years prior^1^. These issues are particularly true for chronic diseases^1^, such as chlamydial disease of koalas, where pathology can be persistent^3,4^ and often not clinically evident^5^. Immunophenotyping, or immune profiling, has been proposed as an effective tool to evaluate the health of wildlife populations and the factors that affect wildlife health. It has the potential to identify protective responses^6^, and predict subsequent effects on population fitness^7^ and disease dynamics^6,8^. Immunological responses can be modulated by diverse internal factors such as sex, age, stress, pathogen burdens^7,9–13^, and external factors such as season, anthropogenic pressures and environmental spatial and temporal features^9,14–17^. Thus, they promise to provide an indicator of real-time impacts of potential disease drivers.

Despite the benefits of immunophenotyping, comprehensive wildlife immunology studies are uncommon, likely due to the complexity in understanding the immune response and the interrelationships that exist with internal or external processes. As a result, most wildlife immunological studies perform separate analyses of each variable. To overcome these challenges, an integrative multi-variate approach assessing the various arms of the immune response in conjunction with host, pathogen and environmental data^6^, would enable more confident identification of biologically relevant immunological variations and mechanisms of complex systems^6,18,19^ and profiles characteristic of different infection and health states^14^. While classical and cross-species techniques of measuring immunological responses such as bacterial killing and complement assays are frequently used and demonstrate value to assess immune function, they represent only one or limited components of the immune system^20^. Advances in gene expression technologies, specifically RT-qPCR and mRNA analysis^21^, have facilitated more extensive investigations of the immune system and improved our understanding of the immune response^22^. For example, multivariate immunophenotyping of innate and adaptive immunity performed in Australian Sealion pups (*Neophoca cinerea*) allowed the interactions of IL-6, B lymphocytes, and antibody responses to be understood in the context of pup development and clearance of hookworm infection^14^.

The koala, *Phascolarctos cinereus*, is an ideal candidate in which to explore the utility of a multi-variate immunological approach to evaluate impacts on health. Koalas face numerous threats to population survival^23–25^, and limited multi-variate immune studies have explored individual associations with environmental, pathogen and host data^12,15^. The koala is listed as endangered within the Australian Capital Territory, Queensland, and New South Wales (NSW)^26^, due to habitat destruction, dog attacks, bushfires, vehicle collisions, climatic change and disease. The threat from disease is primarily due to *Chlamydia pecorum* induced infertility^23–25^. Chlamydiosis in koalas can be persistent^3,4^ and not all disease is clinically evident^5^, posing challenges to interpreting impacts of disease drivers on outcomes in the field. Chlamydiosis can manifest as either subclinical or clinical keratoconjunctivitis, cystitis^5,27^, renal disease^28^ and reproductive disease within male^29–31^ and female koalas; the latter comprising development of para-ovarian cysts, uterine inflammation and fibrosis^28,32,33^. Concomitant infections with other pathogens have been associated with chlamydial infection and disease outcomes, including Koala Retrovirus (KoRV)^34,35^; and co-infection with trypanosomes^36–38^, and Phascolarctid gammaherpesvirus types 1 (PhaHV-1) and 2 (PhaHV-2)^39,40^. Numerous studies have also been conducted to examine associations between stress in the koala and environmental variation such as climatic conditions, seasonal changes, fire and clearing of land^41–43^, but studies examining immunological impacts of these changes are lacking. Such studies are needed as neuroendocrine and immune systems can modulate each other’s function^13,44^. For example, as a portion of leukocytes possess receptors for cortisol and other endocrine molecules^45,46^, cytokine activity can be altered by these molecules^47^. How immunological responses may be modulated by endocrine molecules may depend on whether stress events are acute or chronic, as demonstrated in varying upregulation of pro-inflammatory cytokines such as IL-6, across acute and chronically stressed humans^48–50^.

Appropriate responses to chlamydial infection require a finely balanced innate and adaptive immune response, including cell-mediated (Th1 and Th17) and humoral (Th2) pathways^51–53^. The innate response is required for the initial recognition of the bacterium, initiating inflammatory responses, eliminating infected cells, and helping direct the subsequent, more-specific adaptive responses^53^. Within the adaptive response, the Th1 is proposed as necessary for a protective response^54^ by inhibiting chlamydial growth^55^, and an insufficient response can result in chronic inflammation and fibrosis^56^. Th17 responses, characterised by the cytokine IL-17A, promote Th1 responses^57^, host defence^58^, and clearance^59^; induce memory immunity; and prevent development of reproductive pathology in female mice^60^. Th2 responses have antagonistic effects towards Th1 pathways^61^, which is necessary for regulating inflammatory responses^52^. Across the innate and adaptive arms, overly florid responses in response to infection with *Chlamydia* can however result in damage to the host^51,52^ as well as chronic infection^52^. The function and activity of innate and adaptive elements can be influenced by a range of pathogen and host physiological and genetic traits^61^. The relative importance of co-infections and other host, pathogen and environmental features in shaping these responses and chlamydial disease outcomes in koalas is still not understood.

This study evaluates a novel multi-variate immunological approach to determine the degree of immunological variation in the koala, exposed to differing geographical, climatic and co-infection conditions. We aimed to evaluate whether innate and adaptive responses differed between the Liverpool Plains (LP) and the Southern Highlands to South-west Sydney (SHSWS) koala populations and within the LP population over three time points. Variations in immunophenotype were examined for association with co-infection parameters, such as KoRV transcription, and *C. pecorum,* PhaHV-1 shedding and blood trypanosome infection, and clinical data such as urogenital inflammation and age, stress indicators. The approach used in our study is novel in that we examine immune variation across both the innate and adaptive arms of the immune system in a multi-variate analysis, in association with climatic, co-infection and host features. Relative to univariate studies, it enables our study to identify more biologically meaningful interpretation of immunological variation.

## Materials and methods

### Ethics

Samples were collected in the LP with approval by the University of Sydney Animal Ethics Committee (#2018/1442), (2019/1547) under Scientific License SL102331 and within the SHSWS population with approval by the NSW Department of Planning and Environment Animal Ethics Committees (#2015–2016/150203/03), (#2017–2018/161206-01) and (#2020–2021/201020-04) under NSW National Parks and Wildlife Service Scientific License SL101508.

### Description of study populations

The LP study site comprised a 15 km radius from − 31.159855, 150.118456, within the Liverpool Plains of northern NSW, where koalas exist across a fragmented landscape consisting of agricultural and other privately owned land^62^. This population faces periodic climatic pressures such as heat waves and drought^63^, as well as a high prevalence of chlamydial disease^64^ associated with infertility and population decline^65^. The other population: SHSWS, is located across the Southern Highlands within the Wingecarribee Local Government Area and South-west Sydney within the Wollondilly Local Government Area. In contrast, this population contains a variety of habitat types ranging from dense continuous forest through to forest-urban landscape and a lower prevalence of chlamydial infection and clinical disease and greater fecundity have been recorded (unpublished data).

### Sample collection

Samples were collected over 2019–2021 as part of larger ongoing population health studies. Within the LP, 108 samples were collected from 90 koalas across three sampling time points: time point A (July of 2019, n = 38), time point B (January 2020, n = 26) and time point C (September 2020, n = 44) (Table 1). This included 33 repeated sample sets of 15 koalas. Within the SHSWS, 41 koalas were sampled across two time points: time point D (September 2019, n = 9) and time point E (February, March and April 2021, n = 32), with no repeated sampling of individuals (Table 1). Mean monthly maximum and minimum temperatures for the month prior to sampling, and the average rainfall (mm) in the three months prior to sampling, were obtained from landholder data for the LP site as well as the Bureau of Meteorology (BOM)^66^ (Table 1). Wild koalas were captured from trees using the ‘noose and flag’ technique^67^ and sedated with alfaxalone (Alfaxan, Jurox; 1.8–2mg/kg) for sample collection. The following were recorded: Body condition score (BCS), ranging from 1 (emaciated) to 5 (excellent); sex; body mass (kg); and age class (A to I), estimated by tooth wear as in Gordon, (1991)^68^. Among females, the presence of back or pouch young was recorded. At each sampling, swabs were collected from each conjunctiva and from the urogenital sinus or penile urethra. However, only the urogenital swabs were considered in our study due to the low prevalence of ocular chlamydial infection (6.6% prevalence across time points A, B and C and 4.3% prevalence across time points D and E). The severity of chlamydial clinical signs at the urogenital site was graded by a Wet Bottom Score (WBS) based on Griffith, (2010)^69^; each koala was graded across a scale of 0–10, whereby we considered a grade of 0–0.5 to represent an absence of clinical disease and grades 1–10 to represent clinical disease. WBS grading was performed by the same researchers across sampling field trips, with reference to a photographic standard and descriptive criteria. Approximately 1ml of blood was collected into an EDTA tube for measuring packed cell volume (PCV), total plasma protein (TPP) and haematological analysis. To measure PCV, a microhematocrit capillary tube was filled to approximately 80% of its length with EDTA blood, sealed with plasticine and centrifuged (12,000 rpm) for 3 min. The PCV was determined by placing the capillary tube on a microhematocrit line reader. TPP was determined using a refractometer. To determine the absolute counts of neutrophils and lymphocytes (× 10^9^/L), blood smears were made from the EDTA blood and 100µl of EDTA blood separated into a 1.5ml eppendorf, fixed in Streck Cell Preservative (Streck Cell Preservative, Omaha, United States) at a 1:10 ratio, and stored at 3°C for subsequent differential analysis in the lab. Total white cell counts were determined by Sysmex Hematology Analyzer (XN-1000 series) and differentials were determined from the blood smears by light microscopy. Within 8 h of collection, additional EDTA blood was centrifuged (4000 × g), and the white cell fraction (buffy coat) aspirated, stored in liquid nitrogen and then subsequently fixed in RNA*later*™ solution (Invitrogen™) to minimise risk of freeze/thaw artefacts and maximise consistency with SHSWS samples. Within the SHSWS, EDTA Buffy coats were only collected from those animals sampled in 2019 and EDTA whole blood from animals collected in 2021. Of the EDTA whole blood, 200 µL was fixed in RNA*later*™ solution for extraction of RNA (Invitrogen™) and stored at − 20°C until processing.

**Table 1:** Sample sizes of koalas in each time point analysed by PCA and average maximum, minimum temperatures (°C) of the month prior to sampling and the average rainfall (mm) of the preceding 3 months prior to each time point across time points A, B and C in the LP and D and E in the SHSWS.

Population and time point	Total sample size of buffy coat/whole blood (n)	Sample size (n) in PCA	Number of females	Month prior to sampling	Average maximum temperature (°C)	Average minimum temperature (°C)	Average rainfall (mm)
Liverpool Plains/time point A	38	28/38	17/28	June 2019	18.9	3.5	40
Liverpool Plains/time point B	26	15/26	6/15	Dec 2019	37	17.9	73
Liverpool Plains/time point C	44	29/44	14/29	Aug 2020	18.2	2.1	91
SHSWS/time point D	9	7/9	5/7	Aug 2019	13.2	2.4	98.2
SHSWS/time point E	32	29/32	12/29	Jan, Feb, March 2021	22.43	18.9	306.4

Data was obtained from landholders as well as the Australian Government Bureau of Meteorology (BOM)^66^.

### Extraction of RNA and analysis of immune, co-infection and stress markers using NanoString technology

The RNA from buffy coat and whole blood samples was extracted using the RiboPure™ RNA Purification Kit, Blood (Invitrogen, #AM1928). Briefly, each sample was centrifuged, the RNAlater supernatant aspirated, and the RNA extracted from the cell fraction and stored at − 80°C until further processing. A spectrophotometer (Nanodrop 1000) was used to quantify nucleic acid in each sample prior to analysis by a custom NanoString PlexSet at the Ramaciotti Centre for Genomics, University of New South Wales, Sydney, Australia. The following numbers of samples were not screened by NanoString due to inadequate RNA quantity: time point A, 4/38; time point B, 2/26; time point C, 4/44 and time point E, 1/32 samples. To represent innate and adaptive arms of the immune response, including both humoral and cell-mediated responses, as well as the concurrent stress and co-infection traits of the individuals, the NanoString panel included 35 targets comprising innate and adaptive immune markers (CD3, CD79b, CD4, CD8*β*, IL-10, IL-12A, IL-17A, IL-18, IL-1β, IL-22, IL-4, IL-6, IL-8, IFN-*y*, MHCIUA, *Phci*DAB, *Phci*DBB, TLR2, TLR4, TLR7 and TNF*α*); co-infection targets (KoRV-A *env*, KoRV-B *env*, KoRV-D *env*, KoRVenvCKS17 and KoRV *pol;* and Trypanosome species *T. irwini, T.gilletti, T.copemani*); a stress pathway gene (FKBP5), that encodes FK506 binding protein 51, a co-chaperone protein and mediator of glucocorticoid receptor (GR) function and activity^70^; and four housekeeping reference genes representing high to medium expression levels (GAPDH, ACTB, Stx2, Nckap1)^71,72^. To account for any bias or differences that might arise between buffy coat and whole blood samples, the expression ranges of all 35 targets including housekeeping genes were validated between the buffy coats and whole blood samples from time points D and E of the SHSWS population, with no differing expression identified between sample types.

### Bioinformatic analysis of NanoString raw data

The raw Fastq RCC files generated from NanoString were analysed in NanoString NSolver 4.0 software (NanoString Technologies). The raw copy number counts of mRNA of targets were normalised against the housekeeping genes, without a threshold set. Normalisation allowed for comparison of the relative expression of targets between samples. Briefly, normalisation was performed in NSolver using the following formula (Housekeeping average geometric mean of the row / Housekeeping average geometric mean of individual well) to generate a unique normalisation factor for each individual well. This normalisation factor was then multiplied by the raw counts of each target for each animal. Following normalisation, targets with > 50% of mRNA counts above detectable limits, across all samples and all 5 time points, were included in further analysis. Among these, samples with mRNA counts below the Limit of Quantification (LOQ) for the assay were still included in the analysis as the error likely within this range was considered negligible in comparison to the amplitude of variation overall. Some rejected targets were subsequently evaluated for the full sample set using RT-qPCR if previously published assays were available (see below).

### Real-time qPCR of immune targets: CD4, CD8*β*, IFN-*y*, IL-10 and IL-17A

Due to limited detection by NanoString, the relative expression of CD4, CD8*β*, IFN-*γ*, IL-10 and IL-17A*,* were determined using previously developed and validated qPCR assays^71,73–75^. The target GAPDH^71^, was included as a housekeeping gene. Briefly, for removal of genomic DNA, the extracted RNA was treated using the DNase I, RNase-free kit (1U/µL) (Thermo Scientific™, #EN0521). cDNA synthesis was then performed on the DNase treated RNA using the RevertAid First Strand cDNA synthesis kit (Thermo Scientific™, #K1622) with a Revertaid negative included for each sample to confirm an absence of genomic DNA. PCR efficiencies for each assay were between 90–100% and the inter-assay variance was less than 5% across all targets. Reactions were made to a final volume of 20 µL consisting of 10 µL of SYBR Green Supermix (SsoAdvanced™ Universal SYBR® Green Supermix—BIORAD, 300 nM of each primer, 6.8 µL of dH_2_O and 2 µL of DNA with samples run in duplicates. PCR cycling conditions were a two-step PCR of 40 cycles consisting of an initial denaturation (3 min at 95°C), denaturation (15 s at 95°C) and combined annealing and extension step (30 s at 59°C) for 40 cycles with a melt curve between 55°C-95°C with 5 s stops. The relative expression of each target was then determined using the ΔCt method (Cq average gene of interest ratio − Cq average housekeeping gene ratio)^76,77^. For more intuitive interpretation, the ΔCt was then inverted to 1/ΔCt to make a high value indicate high expression and a low value indicate low expression. Samples with an absence of quantification for the qPCR were excluded from further analysis.

### Quantification of urogenital shedding of *C. pecorum* and PhaHV-1

DNA was extracted from urogenital swabs using the MagMAX™ CORE Nucleic Acid Extraction Kit (ThermoFisher Scientific). To determine the chlamydial shedding at the time of sampling, an established probe-based multiplex qPCR assay was used, targeting the beta-actin mRNA as a housekeeping gene, chlamydial genus 23S rRNA and *C. pecorum ompB* gene^78^. *C. pneumoniae* was not tested for detection in the swabs given its low prevalence on a population level^79^. Other targets such as Mycoplasma and Ureaplasma included in the assay by Hulse, et al., (2018)^78^, were also not included due to their unclear clinical significance. PCR reactions were made to a final volume of 20 µL consisting of 10 µL of SensiFAST™ Probe No-ROX (× 2), 400 nM of each primer, 200 nM of each probe, 4.4 µl of dH_2_O and 2 µL of DNA. Cycling conditions consisted of an initial denaturation (2 min at 98 °C), then 40 cycles of denaturation (15 s at 98 °C) and a combined annealing and extension step (30 s at 58 °C). Samples were run neat (undiluted) and diluted (1:10) to account for inhibitors with shedding considered present if amplification was present across both the 23S and *ompB* targets and shedding absent if amplification was not present across these targets. The average Ct value of the neat *ompB* target was subsequently used for further statistical analysis and if inhibitors were present, the 1:10 value was converted to a neat value based off a 3.3 cycle difference per tenfold dilution in a PCR with 100% efficiency. For the detection of PhaHV-1 in urogenital swabs, an established qPCR assay was used^80^. Briefly, samples were run in duplicate, and each PCR reaction was made up to final volume of 20 µL, consisting of 10 µL of SYBR Green Supermix (SsoAdvanced™ Universal SYBR® Green Supermix—BIORAD), 250 nM of each primer, 7 µL of dH_2_O and 2 µL of DNA. PCR cycling conditions were a two-step PCR of 40 cycles consisting of an initial denaturation (3 min at 98°C), denaturation (10 s at 98°C) and a combined annealing/extension (30 s at 56°C) with a melt curve of 81°C indicating a specific product. A positive control (synthetic plasmid control at 1 × 10^3^ concentration) and No Template Control (NTC) were included in each PCR run with PCR efficiencies ranging between 90–100% and the inter-assay variation being less than 5% across both targets. The average Ct value for PhaHV-1 was used for further statistical analysis.

### Principal components analysis (PCA) and immunological changes between populations

To visualise initial distributions and patterns of expression of the immune and stress genes and co-infection loads across time points A, B, C, D and E, box and whisker plots were created using R^81^. The variables that yielded quantifiable values above the detection limits in more than 50% of the samples (Nanostring, 9/21 immune variables; qPCR, 4/5 variables), were selected for inclusion in the PCA (CD3, CD79b, IL-1β, IL-6, IL-8, *Phci*DAB, *Phci*DBB, 1/ΔCD4, 1/ΔCD8*β*, 1/ΔIFN-*γ*, 1/ΔIL-10), along with absolute counts of neutrophils and lymphocytes (× 10^9^/L), to ascertain whether the variation in cytokine expression, especially low cytokine gene expression relative to housekeeping genes, might be attributed to elevated neutrophil counts.

Before conducting the Principal Components Analysis (PCA), a Spearman's correlation analysis was performed in R^81^, to investigate potential correlations among the variables with all the continuous variables to be input in the PCA. A total of 108 observations across the LP and SHSWS with no missing data across the 13 immune variables were used in the analysis, which included LP time point A (n = 28), time point B (n = 15), time point C (n = 29) and SHSWS time point D (n = 7), time point E (n = 29) (Table 1). Proportion of females were: time point A (17/28), time point B (6/15), time point C (14/29), time point D (5/7) and time point E (12/29). No females were identified to be carrying joeys across time points A, B and C but young were identified in 2/5 females in time point D and 4/12 females in time point E. Within the LP, for individuals sampled at multiple time points, only data from one time point was used in the PCA. Selection of time points for inclusion was dependent on creating the best balance of sample sizes across time points A, B and C. For example, time point B had had a smaller sample size compared to time point A and C and therefore the data within this time point was selected for over the other time points. To visualise the grouping of the immune variables between populations, across time points A, B, C, D and E and sex, biplots for each of these parameters were created from the Principal Components (PCs) explaining the most variation for both PCAs. The PCs that explained the most variation, were subsequently extracted and analysed by a one-way analysis of variance (ANOVA) with the *emmeans* package^82^, to identify whether PC values significantly differed across time points between the populations.

### PCA and immunological changes across timepoints within the LP population

To determine if immune profiles differed among time points within a population, when isolated from the effect of significant between population differences, a second PCA investigating the same 13 immune variables within the LP population across time points A, B and C was performed. As above, prior to performing the PCA, a Spearman’s correlation was performed to confirm that correlations were present among the continuous immune variables within the population. This PCA included a total of 72 observations from time point A (n = 28), time point B (n = 15) and time point C (n = 29). As above, the PCs that explained the most variation were extracted and analysed by a one-way analysis of variance (ANOVA) and the *emmeans* package^82^, to identify whether PC values significantly differed among time points.

### Statistical analysis: repeated measures multivariate analysis of variance (MANOVA) and Paired t-test of recaptured koalas over time points B and C

The expression of the innate and adaptive responses across the longitudinally sampled events of koalas over time points A, B and C in LP was analysed by a repeated measures multivariate analysis of variance (MANOVA) and paired *t*-tests using R^81^. This analysis was performed as a comparison to the PCA of immune variables over time points A, B and C, to determine whether the relative expression of immune targets within the cross-sectional dataset paralleled the longitudinal dataset. Due to a large quantity of missing data across variables in the 15 koalas recaptured over time points A, B and C in LP, a subset of 10 koalas with a more complete dataset from sampling events in time points B and C were analysed in the paired *t*-test analysis. Prior to performing the repeated measures MANOVA and paired *t*-test analysis, the data were checked for normality using Histograms, Kernel Density and q-q plots and the Shapiro–Wilk test. Log and Tukey transformation methods were applied to non-normally distributed variables with the transformation method producing the closest to normal distribution of data being used. If the data for a variable were not normally distributed in one recapture event but normal in the other, the data from both recapture events were transformed. Two repeated measures MANOVAs were run, each incorporating innate and adaptive variables together based off the grouping of innate and adaptive variables from the PCA over time points A, B, C, D and E, to identify whether a significant difference in these two arms of the immune response existed across recapture events. Where the MANOVA were significant, post-hoc paired *t*-tests were performed for each individual innate or adaptive variable across time-points, using Bonferroni adjustment of *p* values to account for Type I errors.

### Fold difference and change in expression of immune variables between time points A, B, C, D and E and MANOVA

Between time points A, B, C, D and E, the relative fold change in expression of immune variables that contributed the most to the PCs identified to significantly differ between time points, was calculated. For immune variables analysed by NanoString, the fold change for variables were calculated by dividing the mean expression in one time point by the mean expression in another time point. For immune variables analysed by qPCR, the 2^−ΔΔCt^ method^76,77^ was applied using the mean ΔCt values for each time point. Between recaptured koalas over time points B and C in the LP, the relative fold changes of expression of individual innate and adaptive variables identified to be significantly different between time points from the MANOVA were calculated. The fold change in immune variables analysed by NanoString was performed as described above. For immune variables analysed by qPCR, the 2^−ΔΔCt^ method was used^76,77^.

### Associations of immunological changes with co-infection, disease and environmental factors

To identify whether transcription of KoRV-A *env*, KoRV-D *env*, KoRVenvCKS17 and KoRV *pol* differed significantly among all time points A, B, C, D and E across both populations, a one-way ANOVA was used if variances of expression were equal across time points, followed by Tukey’s HSD post hoc tests within the emmeans package^82^; or a Welsh and Brown-Forsythe test if variances unequal. Across the populations, we were unable to explore associations between immune profiles with KoRV transcription, due to the bimodal distribution of KoRV expression between populations (Supplementary Figure S4), or with loads of infection with *C. pecorum* and PhaHV-1, or inflammation at the UGT site, due to the low prevalence of infection with these pathogens in the SHSWS relative to the LP population. We refrained from investigating correlations to age and stress due to substantial population differences between these groups, clear associations could not be established with respect to these factors.

Associations between co-infection parameters, and clinical data such as urogenital inflammation, age and stress with immunophenotype were performed within the LP alone to remove the effect of between population differences. Among time points A, B and C in the LP population, variation of KoRV-A *env*, KoRV-D *env*, KoRVenvCKS17 and KoRV *pol* transcription was evaluated by ANOVA if variances of expression were equal across time points followed by Tukey’s HSD post hoc tests within the emmeans package, or a Welsh and Brown-Forsythe tests if variances were not equal. Individual linear regression models were performed to determine the relationship between the PC values of those variables that explained the most variation in the PCA, and transcription of KoRV-A *env*, KoRV-D *env*, KoRVenvCSK17 and KoRV *pol*, and mucosal loads of *C. pecorum* (shedding) and PhaHV-1. To determine associations between the PC values and the presence and absence of urogenital *C. pecorum*, WBS and age, independent *t*-tests were used. Prior to running the *t*-tests, assumptions of normality and homogeneity of variance of the PCs were checked and data transformed if necessary. Presence/absence of urogenital *C. pecorum* shedding was classed as ‘Shedding’ and ‘Not shedding’, respectively. WBS was grouped into two outcomes: ‘No clinical disease’ (WBS 0–0.5) and ‘Clinical disease’ (WBS 1–4) and included only those individuals identified to be shedding *C. pecorum* at the urogenital site at the time of sampling to ensure only infection-challenged individuals were included. As immune responses can vary across age groups, particularly in older individuals^10^, *t*-tests included age groups representing ‘adult’ (classes D–E) and ‘old’ koalas (classes F–I) with juvenile koalas (classes A–C) excluded due to inadequate sample size. PCs were first evaluated for normality and homogeneity of variance, and transformation performed if necessary.

One-way ANOVAs followed by Tukey’s HSD post hoc tests using the *emmeans* package^82^, was performed to identify differences in PCV, TPP and transcription of FKBP5 across time points A, B and C if variances of expression were equal. Individual linear regression models were then performed to determine the relationship between the PC values of the PCs which explained the most variation in the PCA, and PCV, TPP, and transcription of FKBP5. Linear regression models were also run to determine whether there was any relationship between transcription of FKBP5, and PCV or TPP.

## Results

### Assay performance of immune and stress markers using NanoString and qPCR

The immune targets IL-12A, IL-17A, IL-18, IL-22, IL-4, MHCIUA, TLR2, TLR4, TLR7 and TNF*α* were detected by NanoString in less than 50% of samples and so were not included in analyses, leaving CD3, CD79b, IL-1β, IL-6, IL-8, *Phci*DAB and *Phci*DBB included. qPCR screening enabled addition of CD4, CD8*β*, IFN-*γ*, IL-10, but IL-17A remained excluded due to inadequate detection.

### Detection and quantification of loads of co-infecting pathogens: KoRV, *C. pecorum*, PhaHV-1 and trypanosomes

By NanoString, KoRV-A *env*, KoRV-D *env*, KoRVenvCKS17 and KoRV *pol* were detected in all koalas across both populations; KoRV-B *env* gene transcripts were not detected in any sample from either population. Urogenital *C. pecorum* was detected by qPCR in 54/72 LP koalas across time point A (n = 22/28), time point B (n = 9/15), and time point C (n = 23/29); and 10/36 SHSWS koalas across time point D (n = 3/7) and time point E (n = 7/29). Urogenital PhaHV-1 was detected by qPCR in 40/72 koalas from LP across time point A (n = 15/28), time point B (n = 9/15) and time point C (n = 16/29); and 16/36 koalas from the SHSWS across time point D (n = 4/7) and time point E (n = 12/29). Urogenital swabs were not available for one animal in the LP and therefore it was excluded from these analyses. Among the trypanosome species only *T. irwini* was detected, in 15/41 koalas from the SHSWS; no trypanosome RNA was detected in samples from the LP and so trypanosomes were not considered for further analysis.

### PCA and immunological changes between populations

**Figure 1:**
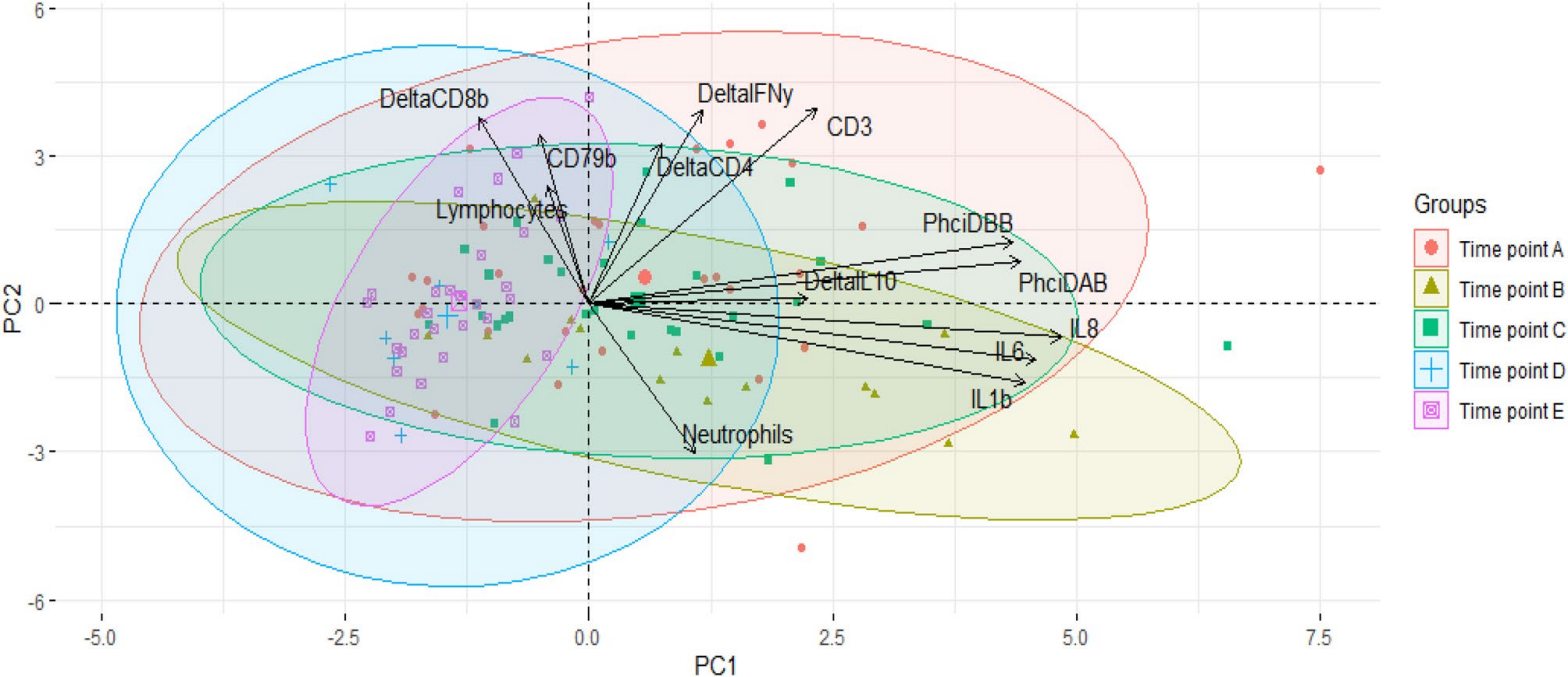
Principal Components Analysis (PCA) biplots of the 13 immune variables across the Liverpool Plains (LP, time points **A**–**C**) and Southern Highlands Southwest Sydney (SHSWS, timepoints **D**,**E**) populations. Immune variables included: Neutrophils, Lymphocytes, DeltaCD4 (1/ΔCD4), DeltaCD8β (1/ΔCD8β), DeltaIFN*γ* (1/ΔIFN-*γ*), DeltaIL10 (1/ΔIL-10), CD3, CD79b, IL1b (IL-1β), IL6 (IL-6), IL8 (IL-8), Phci*DAB*, Phci*DBB*, in koalas sampled over time points A (n = 28), B (n = 15), C (n = 29) in the LP (n = 72 total koalas) and time points D (n = 7) and E (n = 29) in the SHSWS (n = 36 total koalas). The loading vectors for each immune variable are indicated by arrows with the length of arrows representing increasing variance and orientation representing strength of correlations between variables; highly correlating variables are displayed with loadings grouping closer together while less correlated variables position at right angles. Individual koala samples are indicated by the dots on the PCA, with the different colours representing the time point in which these individuals were sampled.

Box and Whisker plot visualisation of the counts of neutrophils and lymphocytes (× 10^9^/L) across all time points A, B, C, D and E, indicated no differences across time points (Supplementary Fig. S1). Across all time points A, B, C, D and E, the Spearman’s rank correlation matrix identified correlations between a substantial number of immune variables. The first two PCs explained a total of 46.9% of the variance with PC1 explaining 26.7% and PC2 explaining 20.2%. Here PC1 represented the innate response with variables IL-8, IL-6, IL-1β, *Phci*DAB, *Phci*DBB contributing the most to this component (Supplementary Table S1). PC2 mostly represented the adaptive response with variables CD3, 1/ΔIFN-*γ*, 1/ΔCD8*β*, CD79b, 1/ΔCD4, and neutrophils, contributing the most (Supplementary Table S1). Biplots suggested differences between the time points in LP and SHSWS populations (Fig. 1) but did not show distinct grouping of immune variables by sex. ANOVA including all time points A, B, C, D and E indicated a significant difference in both innate (PC1: F_(4, 103)_ = [15.21], *p* ≤ 0.001) and adaptive (PC2: F_(4, 103)_ = [2.902], *p* = 0.035) responses. Significant differences in the innate component (PC1) were present between all LP time points to time points D and E within the SHSWS: A vs D (t_(103)_ = 4.041, *p* ≤ 0.001), A vs E (t_(103)_ = 5.248, *p* ≤ 0.0001), B vs D (t_(103)_ = 4.642, *p* ≤ 0.001), B vs E (t_(103)_ = 5.683, *p* ≤ 0.0001), C vs D (t_(103)_ = 4.181, *p* ≤ 0.001) and C vs E (t_(103)_ = 5.497, *p* ≤ 0.0001). No significant difference in PC1 was identified between sampling events within populations. Within the adaptive component (PC2), time points A and B differed significantly (t_(103)_ = 3.367, *p* = 0.009).

### PCA and immunological changes across timepoints within the LP population

Within the LP alone, across time points A, B and C, the first two PCs explained a total of 48.6% of the variance, with PC1 explaining 26.8% and PC2 explaining 21.8% (Supplementary Fig. S2). Here PC1 represented an increase in the adaptive response and a decrease in the innate response with PC2 not representing clear adaptive or innate pathways. In order of contribution, variables 1/ΔCD8*β*, IL-1β, 1/ΔCD4, IL-6, IL-8 and neutrophils contributed the most to PC1 with adaptive variables 1/ΔCD8*β*, 1/ΔCD4 having negative loadings and innate variables IL-1β, IL-6, IL-8 and neutrophils having positive loadings (Supplementary Table S2). The remaining variables did not contribute as strongly to this PC (Supplementary Table S2). There was a significant difference in adaptive-innate balance (PC1) among time points A, B and C (PC1: F_(2, 69)_ = [5.433], *p* = 0.006) with a significantly lower expression of adaptive loci in time point B relative to time point A (t_(69)_ = 3.291, *p* = 0.004). No significant differences between time point A to C (t_(69)_ = 1.217, *p* = 0.447) or between time point B to C (t_(69)_ =  − 2.297, *p* = 0.063) were identified.

Although not detected by post-hoc analysis of cross-sectional data, MANOVA of longitudinal data indicated immune variation between timepoints B and C. Variables in the innate repeated measures MANOVA model included: IL-1β, IL-6, IL-8, *Phci*DAB, *Phci*DBB and variables included in the adaptive repeated measures MANOVA model included: 1/ΔCD4, 1/ΔCD8*β*, CD3, CD79b. The adaptive variable 1/ΔIFN-*γ* was excluded due to missing values across recapture events. Expression of adaptive variables was significantly lower at timepoint B than C (t_(1)_ = 34.532, *p*-value ≤ 0.0001) but innate variables did not differ (t_(1)_ = 0.082, *p*-value = 0.775). Subsequent paired *t*-tests of adaptive variables identified significantly lower expression of CD4 (t_(9)_ =  − 5.970, *p* ≤ 0.001), CD8*β* (t_(9)_ =  − 7.709, *p* ≤ 0.001), CD3 (t_(9)_ =  − 6.25, *p* ≤ 0.001) and CD79b (t_(9)_ = 4.1493, *p* = 0.005) in time point B relative to time point C.

The fold changes in the relative expression of immune variables contributing the most to PC1 and PC2 identified to be significantly different between time points A, B, C, D and E, and adaptive immune variables in the MANOVA across recapture events in time points B and C, are presented in Supplementary Fig. S3 and Table S3, S4.

### Associations of immunological changes with co-infection, disease and environmental factors

Box and Whisker plots indicated marked similarity in distributions for transcription of KoRV (particularly KoRV *pol*), IL-1β and IL-6 within the LP and SHSWS populations and these differed markedly between these populations (Supplementary Fig. S4). Expression of IL-1β and IL-6 and transcription of KoRV, were greater and more variable in the LP samples compared to the SHSWS (Supplementary Fig. S4). Using the one-way Welch and Brown-Forsythe tests due to differences in variance, significant differences in transcription of KoRV-A *env* (F_(4)_ = [5.445], *p* = 0.002), KoRV-D *env* (F_(4)_ = [8.2608], *p* ≤ 0.001) and KoRV *pol* (F_(4)_ = [13.181], *p* ≤ 0.001) were identified across time points A, B, C, D and E. Transcription of KoRV *pol* differed between time point A relative to D (t_(32)_ = 4.34, *p* ≤ 0.001) and E (t_(39)_ = 4.12, *p* = 0.002), between time point B to D (t_(15)_ = 3.73, *p* = 0.015) and to E (t_(16)_ = 3.65, *p* = 0.016) and between time point C to D (t_(32)_ = 5.12, *p* ≤ 0.001) and E (t_(36)_ = 4.95, *p* ≤ 0.001). Remaining post-hoc differences were less consistent; transcription of KoRV-A *env* differed between time point C relative to D (t_(33.7)_ = 3.12, *p* = 0.028) and to E (t_(29.8)_ = 3.59, *p* = 0.010); transcription of KoRV-D *env* differed between time point A relative to E (t_(32.6)_ = 3.330, *p* = 0.017) and between time point C to E (t_(33.6)_ = 4.560, *p* ≤ 0.001). ANOVA identified a significant difference in expression of KoRVenvCKS17 expression among time all points A–E (F_(4, 103)_ = [2.495], *p* = 0.047) with differences present between time points C to E (t_(103)_ = 2.753, *p* = 0.0531).

Within the LP population, among timepoints A, B and C, there was no difference in loads of *C. pecorum* and PhaHV-1 and no association of immune profiles to these or urogenital inflammatory disease or age. ANOVA across time points A, B and C, indicated no significant difference in loads of shedding with *C. pecorum* (F_(2, 51)_ = [2.062], *p* = 0.138) and PhaHV-1 (F_(2, 37)_ = [0.415], *p* = 0.663). The regressions between PC1 and co-infection parameters were not supportive of associations: KoRV-A *env* (R^2^ = [0.002], F_(1,70)_ = [0.113], *p* = 0.738); KoRV-D *env* (R^2^ = [0.073], F_(1,70)_ = [5.556], *p* = 0.021); KoRVenvCSK17 (R^2^ = [0.058], F_(1,70)_ = [4.312], *p* = 0.042); KoRV *pol* (R^2^ = [0.098], F_(1,70)_ = [7.627], *p* = 0.007); loads of *C. pecorum* (R^2^ = [0.002], F_(1,52)_ = [0.115], *p* = 0.7363) and PhaHV1 (R^2^ = [0.006], F_(1,38)_ = [0.247], *p* = 0.622). Similarly, no significant difference in PC1 was identified between those koalas shedding (n = 55) and not shedding (n = 16) urogenital *C. pecorum* (t_(69)_ =  − 1.247, *p* = 0.217); between WBS ‘No clinical disease’ (n = 40) and ‘Clinical disease’ (n = 14) (t_(52)_ =  − 0.157, *p* = 0.876); or between age ‘D-E’ (n = 30) and ‘F-I’ (n = 35) (t_(63)_ = 0.790, *p* = 0.432). Within time point B, PC1 did not differ between age groups ‘D-E’ (n = 5) and ‘F-I (n = 6) (t_(9)_ =  − 0.017, *p* = 0.987).

Expression of the stress pathway gene FKBP5 differed among the three time points (F_(2, 69)_ = [23.42], *p*-value ≤ 0.001), with time point B having significantly greater expression compared to time points A (t_(69)_ =  − 6.783, *p*-value ≤ 0.001) and C (t_(69)_ = 5.132, *p*-value ≤ 0.001) (Supplementary Fig. S5). PCV and TPP also differed significantly among time points A, B and C (PCV: F_(2, 67)_ = [27.84], *p* ≤ 0.001), (TPP: F_(2, 67)_ = [31.49], *p* ≤ 0.001) with a significantly lower PCV and TPP in time point B compared to time points A (PCV: t_(67)_ = 7.181, *p* ≤ 0.001), (TPP: t_(67)_ = 7.714, *p* ≤ 0.001), and C (PCV: t_(67)_ =  − 6.192, *p* ≤ 0.001), (TPP: t_(67)_ =  − 6.414, *p* ≤ 0.001) (Supplementary Fig. S5). Although lower PC1 values (adaptive immunity), TPP, and PCV and greater FKBP5 transcription all occurred in timepoint B, regression analysis supported only very weak relationships between these variables: FKBP5 to PCV (R^2^ = [0.201], F_(1,68)_ = [17.07], *p* ≤ 0.001) and TPP (R^2^ = [0.282], F_(1,68)_ = [26.74], *p* ≤ 0.001); and no relationship between PC1 and FKBP5 (R^2^ = [0.106], F_(1,70)_ = [8.332], *p* = 0.005), PCV (R^2^ = [0.092], F_(1,68)_ = [6.882], *p* = 0.012), and TPP (R^2^ = [0.018], F_(1,68)_ = [1.278], *p* = 0.262).

## Discussion

This is the most extensive study investigating both innate and adaptive immune responses together with climatic, pathogen and host data in the koala. This study demonstrates the utility of a multi-variate immunological analysis to evaluate population health in a wildlife species, across diverse geographical, climatic and co-infection contexts. As an association-based study, the aim was to determine how, and to what degree, immune profiles of koalas changed under these differing conditions. Our multivariate approach covering both the innate and adaptive arms of the immune system, greatly facilitated interpretation of observed changes in immunophenotype and their association with concurrent co-infection and climatic events in a way that would not have been possible using single variate immune markers and disease outcome as measures of host susceptibility. The LP population, a population experiencing drought and a greater retroviral expression and chlamydial disease, had a significantly greater and more variable expression of innate immune parameters compared to the SHSWS. Marked similarity in distributions of KoRV transcription and the innate immune markers IL-1β and IL-6 identifies these immune mediators as candidates for investigation in mechanistic studies of KoRV pathogenesis. Within the LP, significantly greater expression of the stress co-chaperone, FKBP5, and reduced PCV, TPP and transcription of adaptive immune markers coincided with a sustained heat event but limited evidence of correlation between these parameters within individuals, suggests that this variation may arise from multiple mechanisms. Urogenital shedding of *C. pecorum*, PhaHV-1 and urogenital infection status, inflammation and age were not associated with immunological changes, suggesting that immune variation was driven by processes other than, for example, chlamydial inflammation or old age. The outcomes of this study are a novel approach to investigate drivers of wildlife disease and more clearly identified associative relationships to direct mechanistic or replicated field studies to elucidate causative relationships.

The multi-variate immunological approach used in this study was novel in that it provided perspectives and interpretation of immunological variation across the different arms of the immune system that would not have been observed using single variables alone or using disease outcomes as indicators of susceptibility. Given the complexity of the immune response, variations or associations detected in individual immune variables may not be representative of overall pathways being affected, or determine which variations are biologically meaningful^6,18,20^. Past immunological studies of koalas, in relation to KoRV infection^12,15^, have shown variation in individual parameters but interpretation of relevance of these to the performance of the immune response as a whole has been challenging. The use of PCA facilitated a systems consensus approach that more clearly associated changes with the innate or adaptive responses. The use of immunophenotype, including innate and adaptive immune function as an outcome, demonstrated immune changes in real time, which were not evident in external or visual manifestations of disease, due to its chronic and often permanent nature. In koala health surveys, the WBS grading system is utilised as an indicator of inflammation at the urogenital site^69^, however the approach is limited as it is only possible to observe outcomes in animals that have contacted the pathogen and, among those, some outcomes, such as reproductive disease, are not always clinically evident^5^. In addition, due to the nature of chlamydial disease, observed signs may represent either current inflammation or chronic fibrotic pathology of the bladder which may have occurred months to years prior. Use of immunophenotyping appeared to circumvent these issues.

The greater expression of innate immune genes and the similarity in distributions of KoRV, IL-1β and IL-6, highlight the degree of variation between koala populations in different contexts, though the mechanisms involved require further work to elucidate, due to multiple differences between the populations. Koalas within the LP have experienced and continue to endure more extreme climatic conditions such as heatwaves and drought^63^ and also live across a moderately fragmented habitat^62^. At the time of the study, the SHSWS population experienced greater rainfall and more moderate ambient temperatures as shown in Table 1^66^, and koalas appear to live across more continuous habitat in the north of the population. The difference in KoRV expression between the populations might have been related to the north–south cline reported by Blyton et al.^34^ and may or may not be influencing immunophenotypic variation. Alternatively, the differing immune status may have influenced KoRV expression, or the two may be linked by some yet unidentified common factor. Numerous studies of koalas have demonstrated associations between KoRV infection and variable expression of cytokines across Th1, Th2 and Th17-A pathways^12,15^. The similar distributions of innate cytokines IL-1β and IL-6 and KoRV *pol* transcription loads (Supplementary Fig. S4) are of interest considering a previous study in mice where increased secretion of IL-1β was induced by, and enhanced, retroviral infection, replication and persistence in vivo by recruiting susceptible cells to the sites of infection creating a positive feedback loop for retroviral infection^83^. Induction of other cytokines IL-10, IL-6 and IL-8 following infection with KoRV^84,85^ and other retroviruses^86^, has also been described; we found associations with IL-6, some correlation with IL-8 but not IL-10. Given that these findings are based on associative relationships, further mechanistic studies should be performed investigating the directionality and causality of these associations.

Within the LP alone, no association between the decreased expression of the adaptive response to transcription of KoRV during the extreme weather event was identified, which suggests that within in this event, adverse environmental conditions were more important in driving immunological variation than KoRV was, though it appears likely this is manifesting through multiple pathways. The consistent differences between sampling events but weak correlation between expression of FKBP5, PCV and TPP and adaptive variables suggests complex interactions that may involve, for example, heat, hydration, nutrition or exposure to PSMs. The lower PCV (anaemia) and TPP values, and elevated expression of a stress receptor pathway gene FKBP5, occurring in time point B, are all consistent with expected impacts of extreme ambient temperatures. Anaemia has been identified in sheep^87^ and humans experiencing prolonged heat exposure^88^. Erythropoiesis can be reduced under heat stress events due to an increased partial pressure of oxygen in the blood from increased respiratory rates of animals experiencing heat^87^. Although we did not identify any associations between elevated expression of FKBP5 and reduced PCV and TPP and the decreased adaptive response during this event, in humans, increased stress impacts immune function, specifically by increasing inflammation^89^ and expression of innate cytokines: IL-1β and IL-6^48–50^. Koalas in time point B were observed to be in poorer body condition, consistent with strategic decrease in food intake by endotherms in increased ambient temperatures to maintain their body temperature^90^ and reduce diet induced thermogenesis^91^. This however may suppress immune investment, specifically adaptive responses^92^, as initiating and maintaining an immune response requires energy expenditure^93^ and can elevate metabolic rates^93,94^. In a study of leukocyte profiles of bats across the Neotropics, bats at the edge of their distributions had increased variation and quantities of white blood cell counts, likely from chronic stress due to the greater variation in temperature and rainfall experienced at these range limits^17^. Greater ambient temperatures have also been suggested to increase production of secondary metabolites across a number of plant species^95^ and PSMs have been shown to impede koala immune cell function in vitro at concentrations experienced in their plasma^96^. Increased CO_2_ emissions as a result of climate change can also increase concentrations of PSMs such as phenol and tannins^97^. We did not measure PSMs, however we speculate that extended exposure of koalas to increasing PSMs as a result of environmental extremes which are predicted to occur more frequently within Australia^98^, may adversely impact innate and adaptive responses. These findings highlight the need for this study to be replicated across diverse populations, and coupled with mechanistic studies, to evaluate these alternative hypotheses and the thresholds at which these climatic extremes have significant impact on immune function.

It is unlikely that other measured variables such as population differences in the proportions of chlamydial infection and disease, sex, breeding season and age demographics, are associated with the differences in immune function observed as we found no associations in immune variation with these factors within populations. In comparison to the LP, the SHSWS population has lower prevalence of chlamydial infection and disease but, within the LP, immune profiles did not differ with respect to chlamydial shedding or disease. As a systemic immunophenotype was measured in this study, it is possible that it may not reflect local, mucosal, inflammatory responses in response to infection with *Chlamydia* at the UGT site, particularly as the spectrum of acute to chronic UGT disease, were grouped in this study together as ‘Clinical disease’. We consider it reasonable that systemic challenges posed by varying environmental conditions are likely to exert greater effects on systemic immunophenotypes than would more localised mucosal challenges posed by chlamydiosis. Sex and breeding season are unlikely to have affected interpretation of the immunological variation as sampling time points B and E both occurred within the breeding season over November to February and still differed. Within each of the sampling time points, sexes were balanced. Although the upregulation of IL-6 expression observed within time point B coincides with reports of increased expression of this cytokine in December^15^, the expression patterns of the remaining cytokines of the innate and adaptive responses did not correspond to previously reported changes across seasons. Despite that the SHSWS population had a greater proportion of younger animals, no significant differences in immune responses between adults to older koalas were identified within the LP, likely ruling out any age associated impacts.

Overall, the associative relationships identified in our study will guide additional health studies in the koala to prove causative relationships between altered immune function with climatic conditions and co-infection with KoRV. Future studies should apply the multi-variate immunological approach used in our study, to a highly curated longitudinal dataset over multiple time points and populations in association with environmental and co-infection parameters to define causation and establish thresholds for detrimental impacts. In particular, to better define environmental and climatic conditions and their impacts, additional data such as diet composition, leaf moisture^43,99^ and days since last rain^100^ should be collected. Further development of assays for markers identified to be important in chlamydial infection, but which remained below the limit of detection in our study, in particular IL-17A, should be addressed and included for future studies. This may involve using mitogen stimulation assays to upregulate those markers below the limit of detection in baseline samples, or investigation of more abundant pathway targets such as receptor molecules. Including markers representing all arms of the immune system will facilitate more accurate interpretation of immunological variation^6,18,20^.

## Conclusion

Our study provides a novel approach, in that we assessed variation across the various arms of the immune response, to better quantify immunological responses to external stressors, a useful tool in the context of climate change and co-infecting pathogens. Continued changes to Australia’s climate are predicted to have adverse impacts on koala populations^43,101,102^. Koalas are listed by the IUCN as one of the top 10 species to be impacted by climate change^103^, but thresholds for detrimental impact on immune responses and disease susceptibility are yet to be identified for the species. Additional studies assessing immunological responses in koala populations experiencing climatic extremes are therefore needed. The distributions of upregulation of innate cytokines and increased KoRV transcription identified in our study is seminal work required for directing the focus and efforts for future mechanistic studies to identify the directionality of these relationships and how they may alter disease outcomes. The outcome would be an enhanced ability to predict habitat and population viability in the face of continued challenges placed by habitat degradation, climate change and co-infections. The approach used in our study is also transferable to other epidemiological studies of health in other wildlife species.

### Supplementary Information

Supplementary Information.

## Data Availability

The datasets used and/or analysed during the current study available from the corresponding author on reasonable request.
