# Supplementary material for: A novel multi-variate immunological approach, reveals immune variation associated with environmental conditions, and co-infection in the koala (Phascolarctos cinereus)
Source: Sci Rep. 2024 Mar 27;14:7260. doi: 10.1038/s41598-024-57792-7 (PMC10973505; doi:10.1038/s41598-024-57792-7)
Supplement: Supplementary file 1 — Supplementary Information. [file 41598_2024_57792_MOESM1_ESM.pptx]

## Slide 1
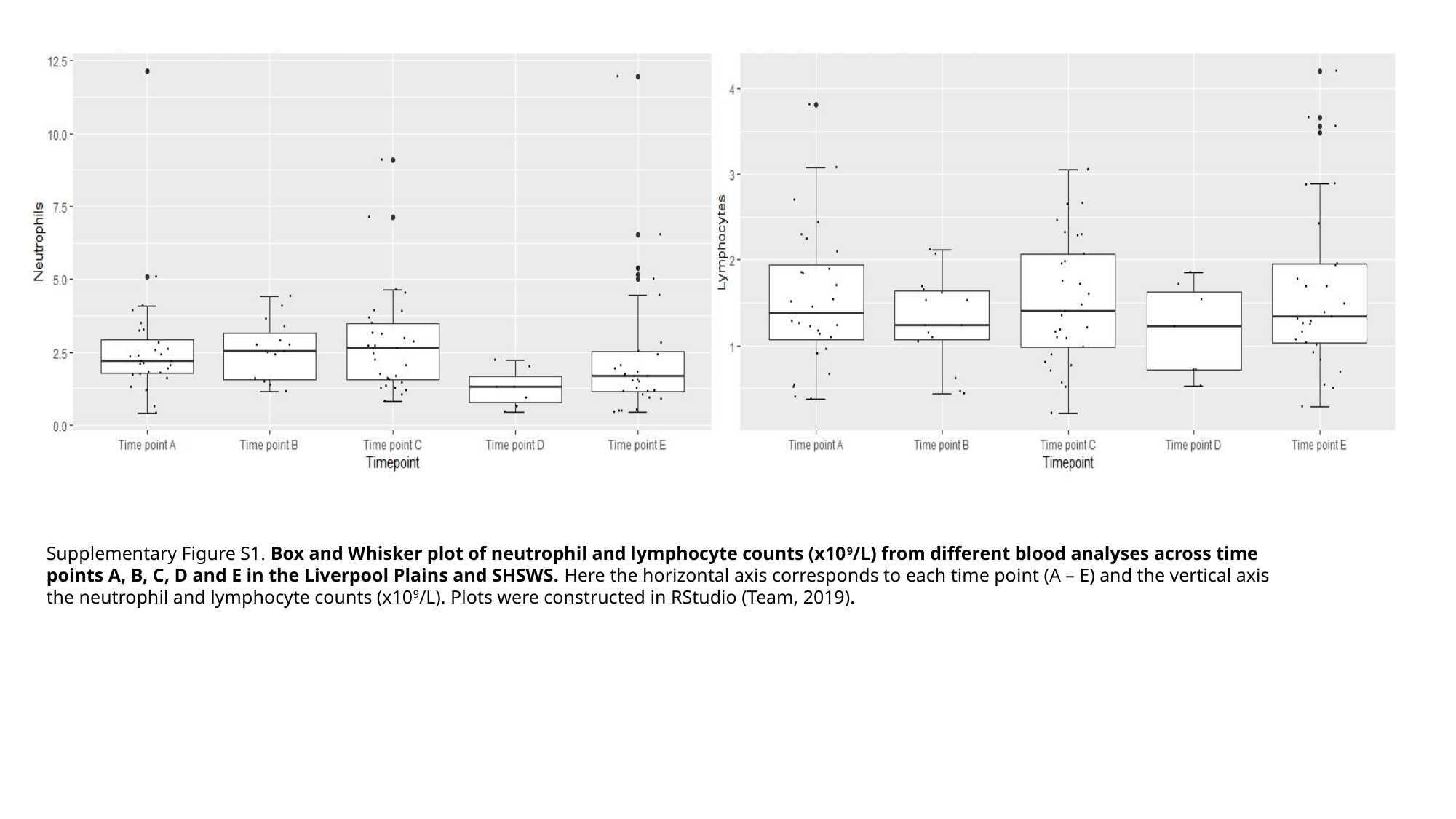

Supplementary Figure S1. Box and Whisker plot of neutrophil and lymphocyte counts (x109/L) from different blood analyses across time points A, B, C, D and E in the Liverpool Plains and SHSWS. Here the horizontal axis corresponds to each time point (A – E) and the vertical axis the neutrophil and lymphocyte counts (x109/L). Plots were constructed in RStudio (Team, 2019).

## Slide 2
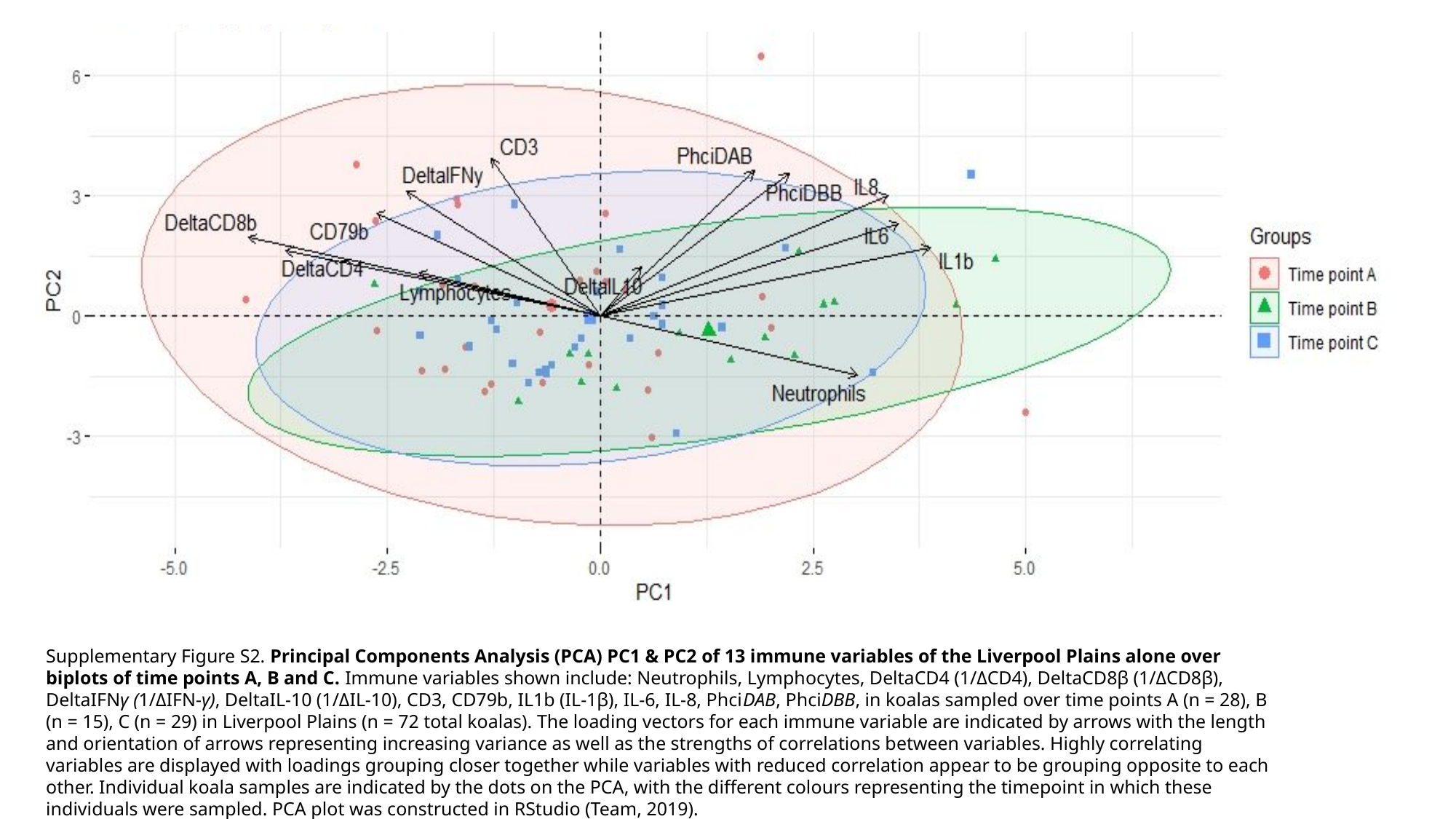

Supplementary Figure S2. Principal Components Analysis (PCA) PC1 & PC2 of 13 immune variables of the Liverpool Plains alone over biplots of time points A, B and C. Immune variables shown include: Neutrophils, Lymphocytes, DeltaCD4 (1/ΔCD4), DeltaCD8β (1/ΔCD8β), DeltaIFNγ (1/ΔIFN-γ), DeltaIL-10 (1/ΔIL-10), CD3, CD79b, IL1b (IL-1β), IL-6, IL-8, PhciDAB, PhciDBB, in koalas sampled over time points A (n = 28), B (n = 15), C (n = 29) in Liverpool Plains (n = 72 total koalas). The loading vectors for each immune variable are indicated by arrows with the length and orientation of arrows representing increasing variance as well as the strengths of correlations between variables. Highly correlating variables are displayed with loadings grouping closer together while variables with reduced correlation appear to be grouping opposite to each other. Individual koala samples are indicated by the dots on the PCA, with the different colours representing the timepoint in which these individuals were sampled. PCA plot was constructed in RStudio (Team, 2019).

## Slide 3
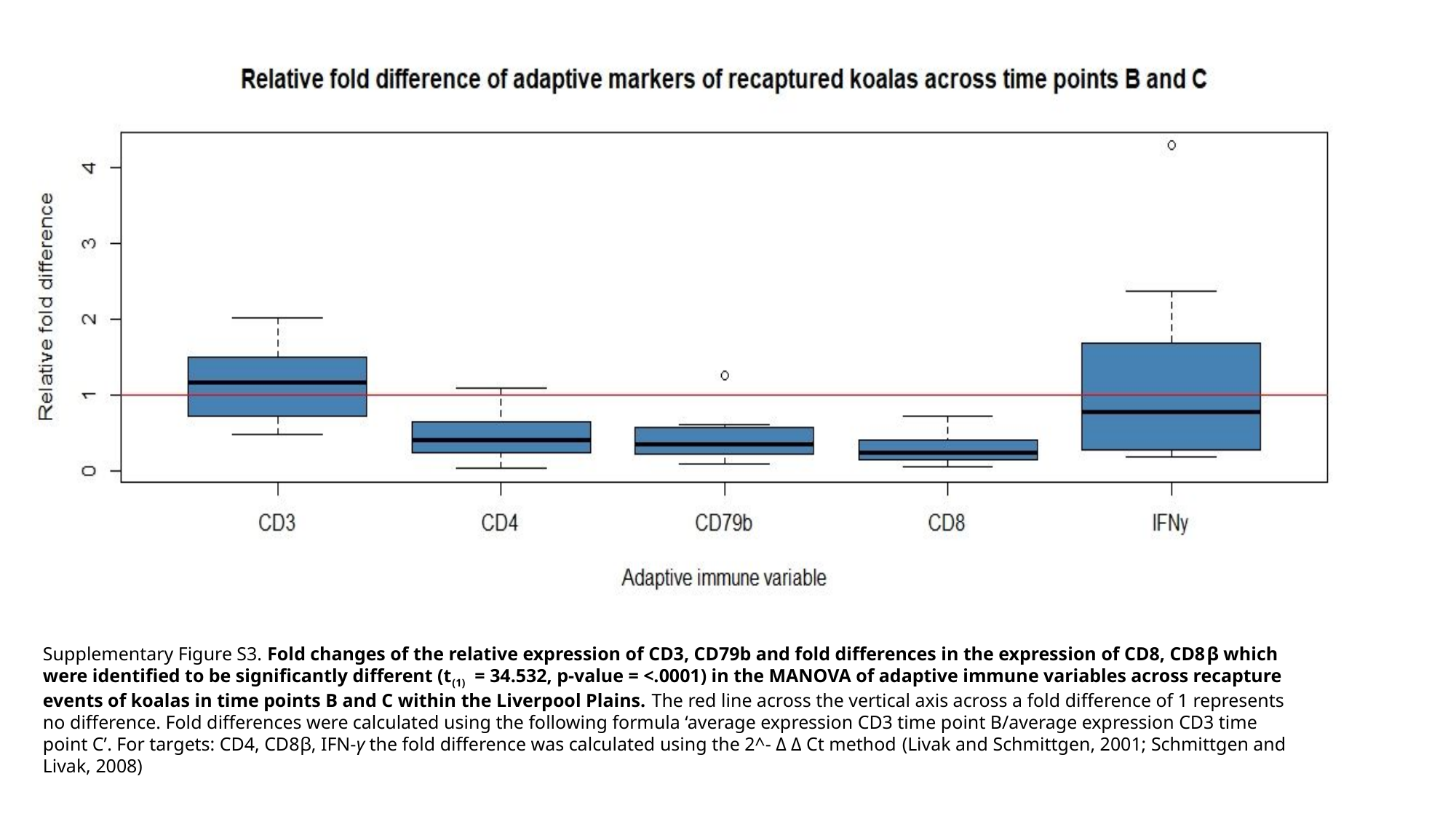

Supplementary Figure S3. Fold changes of the relative expression of CD3, CD79b and fold differences in the expression of CD8, CD8β which were identified to be significantly different (t(1) = 34.532, p-value = <.0001) in the MANOVA of adaptive immune variables across recapture events of koalas in time points B and C within the Liverpool Plains. The red line across the vertical axis across a fold difference of 1 represents no difference. Fold differences were calculated using the following formula ‘average expression CD3 time point B/average expression CD3 time point C’. For targets: CD4, CD8β, IFN-γ the fold difference was calculated using the 2^- Δ Δ Ct method (Livak and Schmittgen, 2001; Schmittgen and Livak, 2008)

## Slide 4
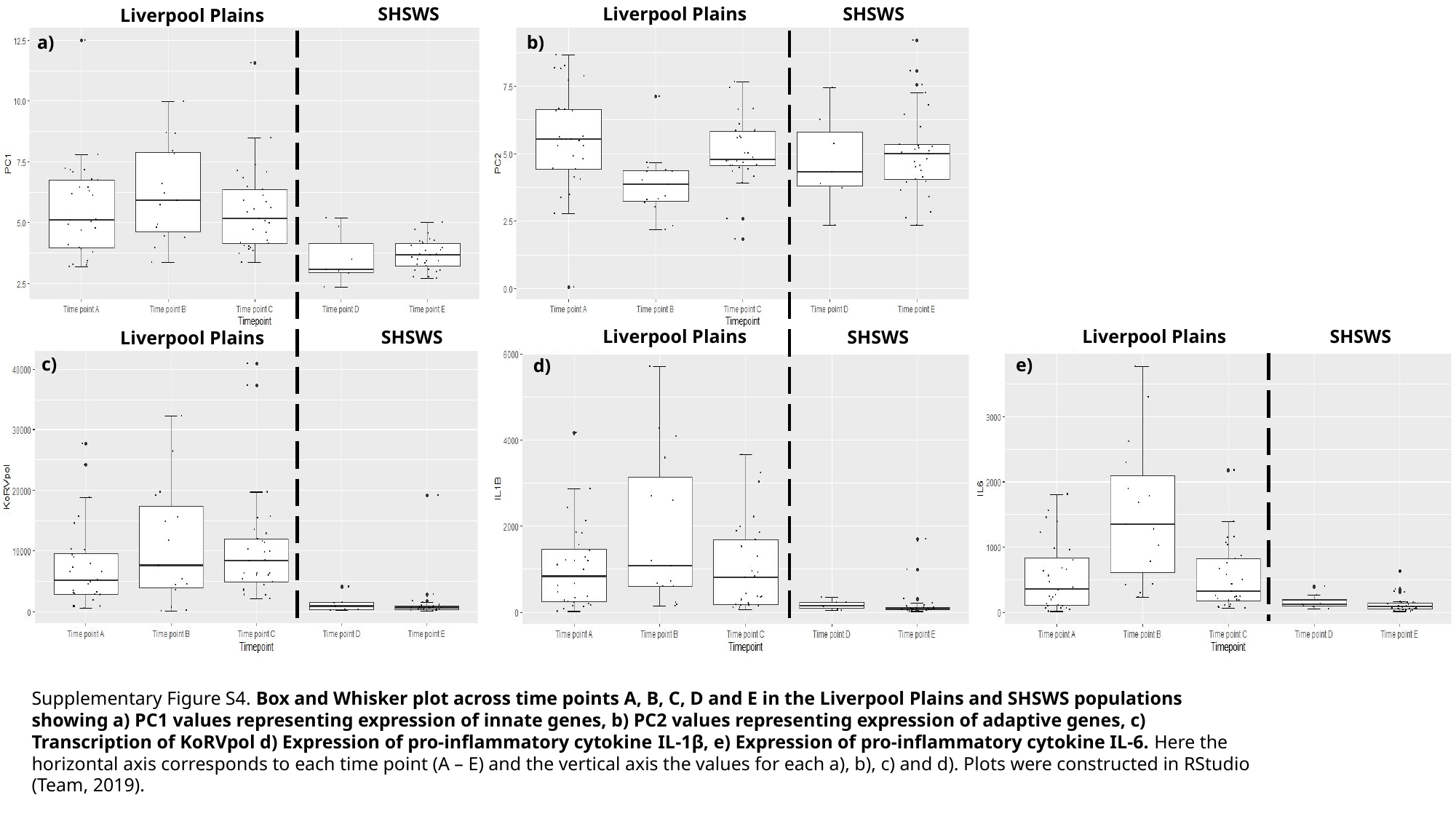

Liverpool Plains
SHSWS
SHSWS
Liverpool Plains
a)
b)
Liverpool Plains
Liverpool Plains
SHSWS
SHSWS
SHSWS
Liverpool Plains
c)
e)
d)
Supplementary Figure S4. Box and Whisker plot across time points A, B, C, D and E in the Liverpool Plains and SHSWS populations showing a) PC1 values representing expression of innate genes, b) PC2 values representing expression of adaptive genes, c) Transcription of KoRVpol d) Expression of pro-inflammatory cytokine IL-1β, e) Expression of pro-inflammatory cytokine IL-6. Here the horizontal axis corresponds to each time point (A – E) and the vertical axis the values for each a), b), c) and d). Plots were constructed in RStudio (Team, 2019).

## Slide 5
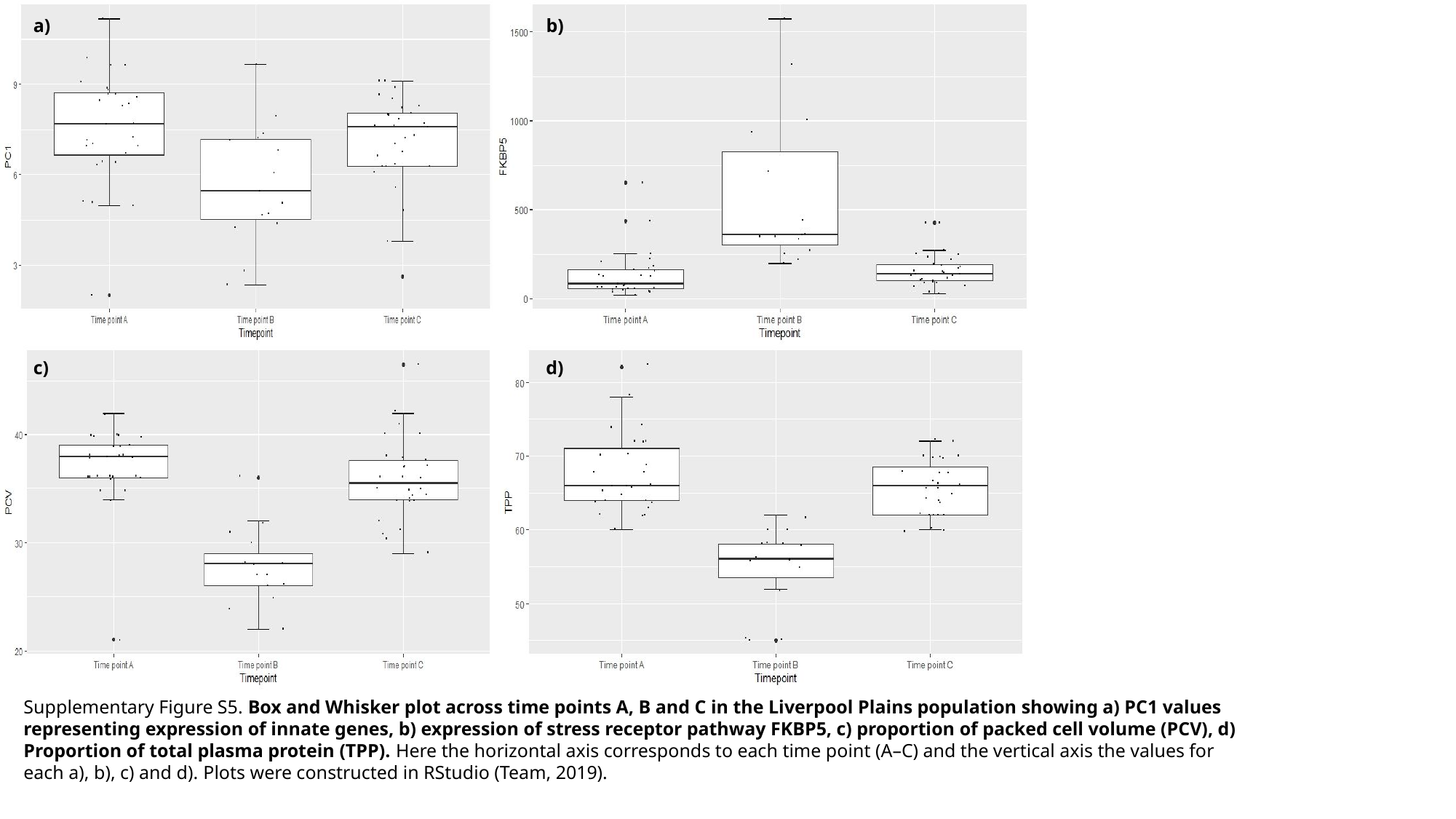

a)
b)
d)
c)
Supplementary Figure S5. Box and Whisker plot across time points A, B and C in the Liverpool Plains population showing a) PC1 values representing expression of innate genes, b) expression of stress receptor pathway FKBP5, c) proportion of packed cell volume (PCV), d) Proportion of total plasma protein (TPP). Here the horizontal axis corresponds to each time point (A–C) and the vertical axis the values for each a), b), c) and d). Plots were constructed in RStudio (Team, 2019).

## Slide 6
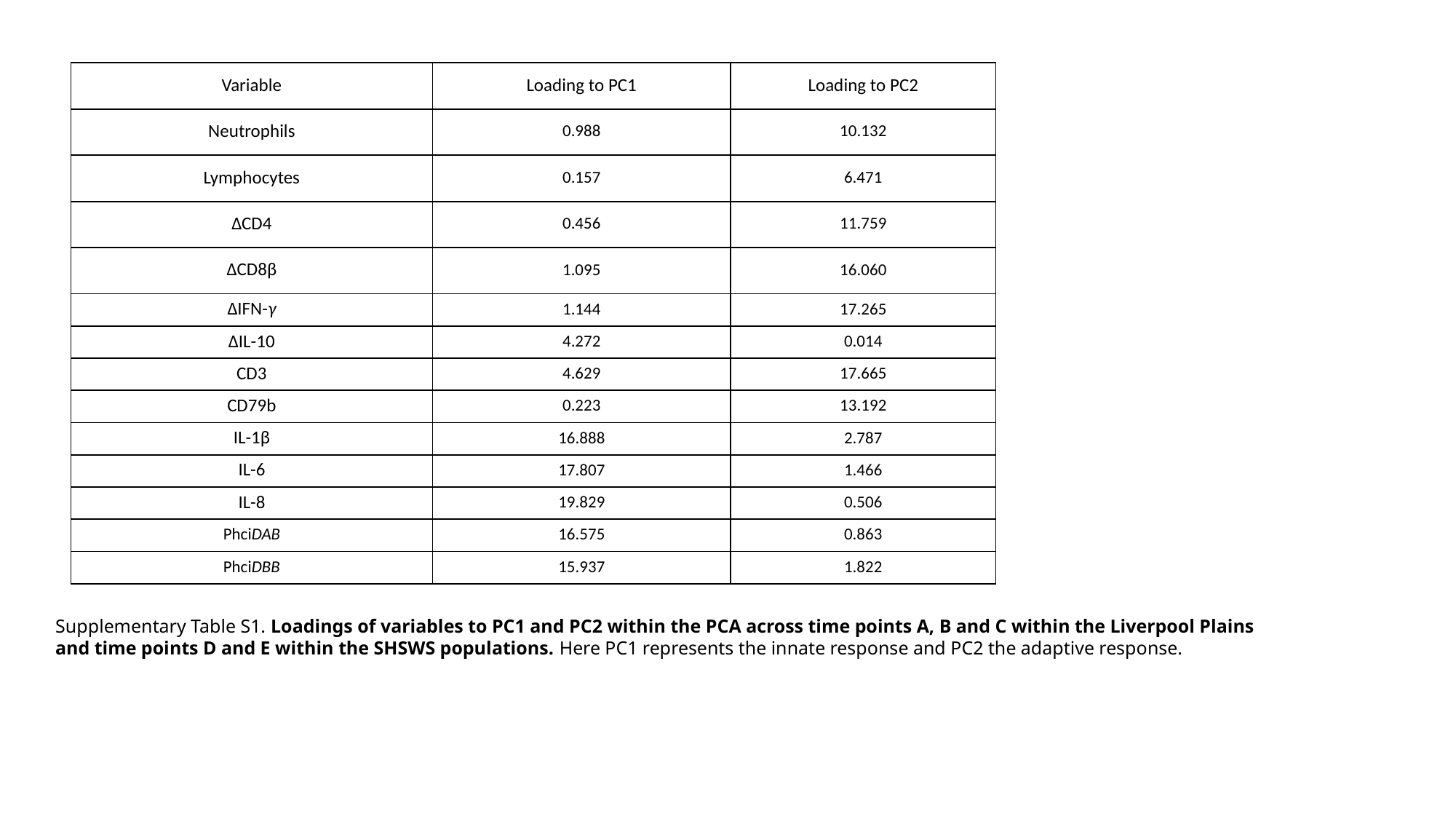

| Variable | Loading to PC1 | Loading to PC2 |
| --- | --- | --- |
| Neutrophils | 0.988 | 10.132 |
| Lymphocytes | 0.157 | 6.471 |
| ΔCD4 | 0.456 | 11.759 |
| ΔCD8β | 1.095 | 16.060 |
| ΔIFN-γ | 1.144 | 17.265 |
| ΔIL-10 | 4.272 | 0.014 |
| CD3 | 4.629 | 17.665 |
| CD79b | 0.223 | 13.192 |
| IL-1β | 16.888 | 2.787 |
| IL-6 | 17.807 | 1.466 |
| IL-8 | 19.829 | 0.506 |
| PhciDAB | 16.575 | 0.863 |
| PhciDBB | 15.937 | 1.822 |
Supplementary Table S1. Loadings of variables to PC1 and PC2 within the PCA across time points A, B and C within the Liverpool Plains and time points D and E within the SHSWS populations. Here PC1 represents the innate response and PC2 the adaptive response.

## Slide 7
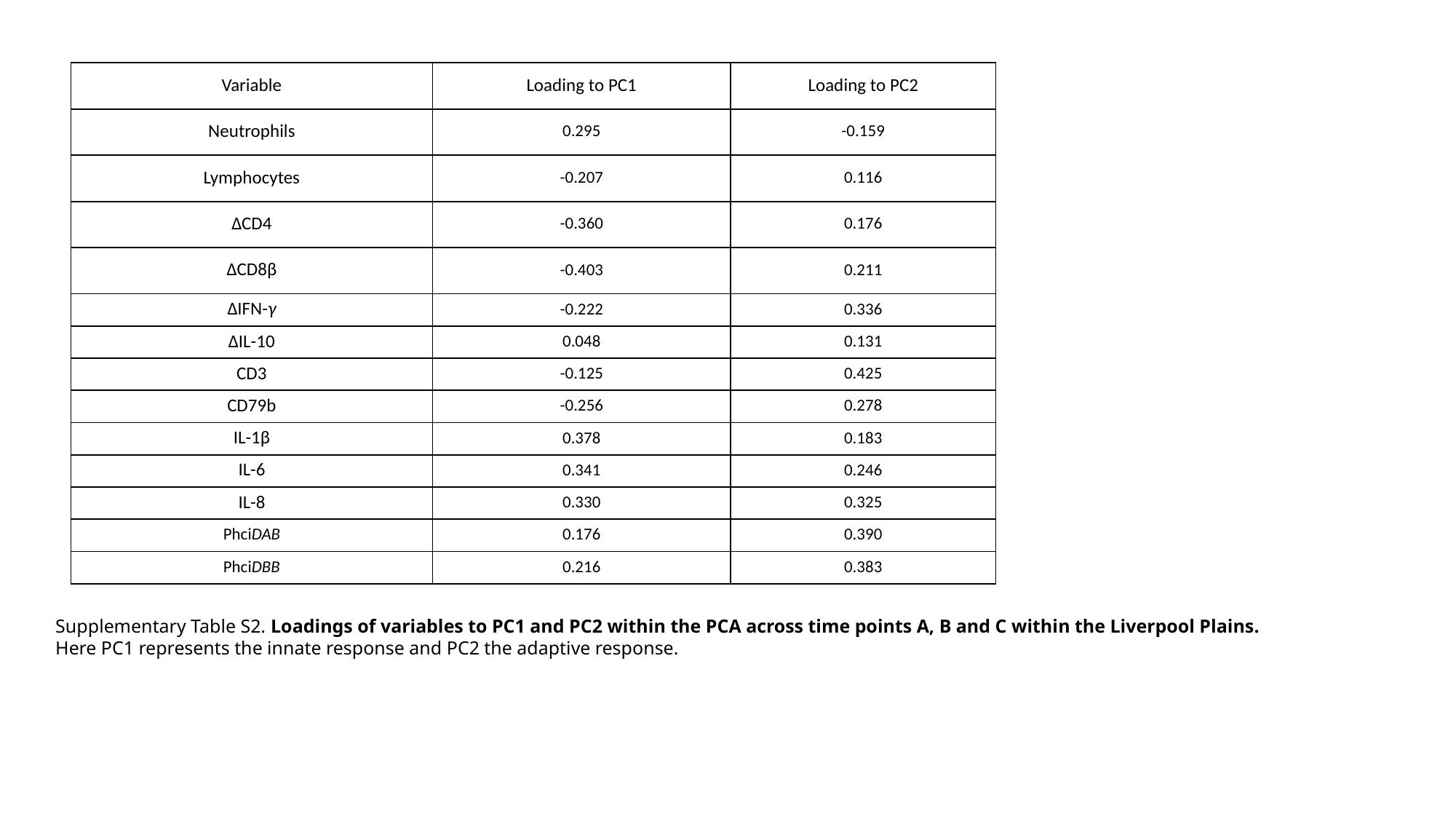

| Variable | Loading to PC1 | Loading to PC2 |
| --- | --- | --- |
| Neutrophils | 0.295 | -0.159 |
| Lymphocytes | -0.207 | 0.116 |
| ΔCD4 | -0.360 | 0.176 |
| ΔCD8β | -0.403 | 0.211 |
| ΔIFN-γ | -0.222 | 0.336 |
| ΔIL-10 | 0.048 | 0.131 |
| CD3 | -0.125 | 0.425 |
| CD79b | -0.256 | 0.278 |
| IL-1β | 0.378 | 0.183 |
| IL-6 | 0.341 | 0.246 |
| IL-8 | 0.330 | 0.325 |
| PhciDAB | 0.176 | 0.390 |
| PhciDBB | 0.216 | 0.383 |
Supplementary Table S2. Loadings of variables to PC1 and PC2 within the PCA across time points A, B and C within the Liverpool Plains. Here PC1 represents the innate response and PC2 the adaptive response.

## Slide 8
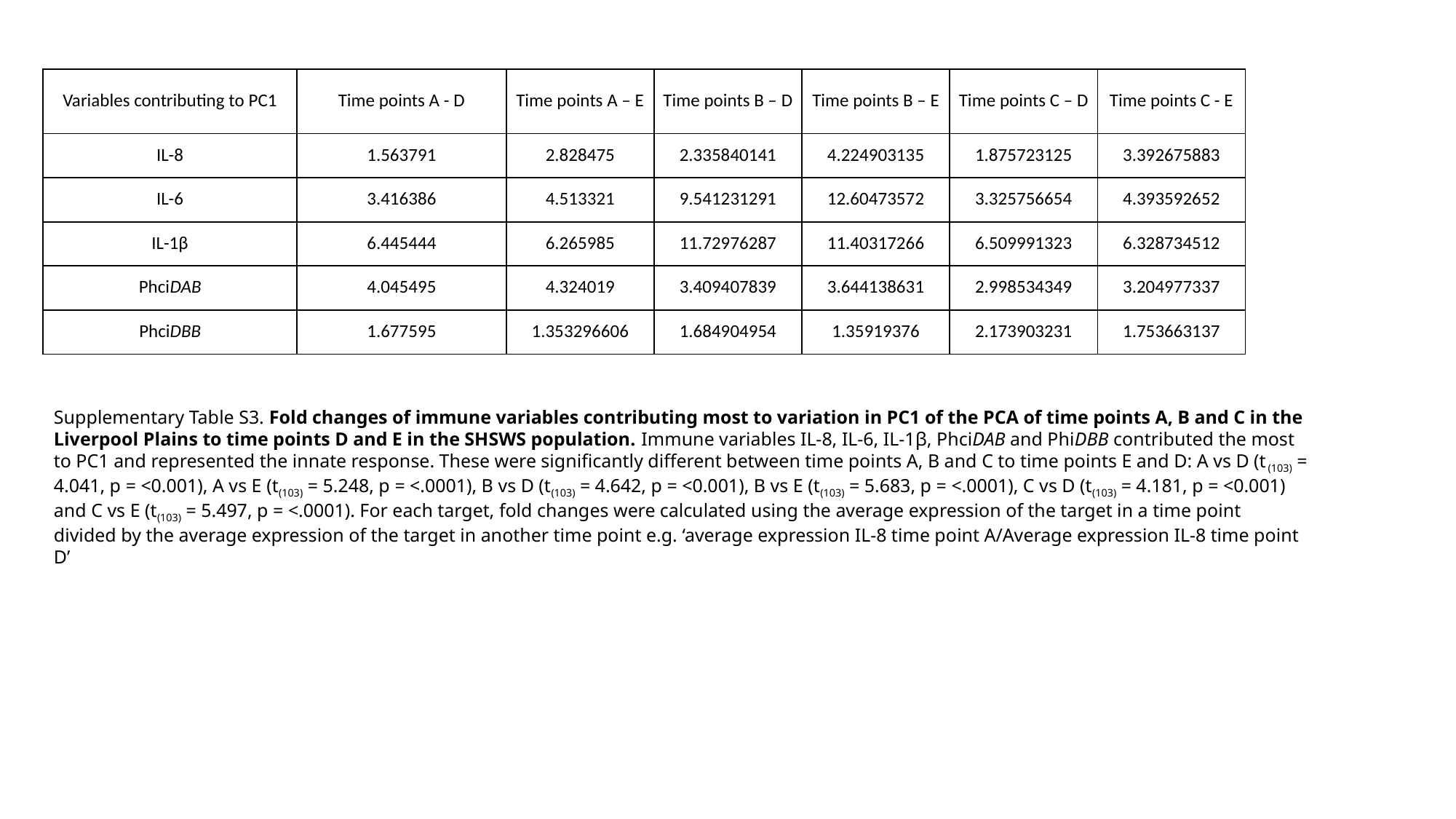

| Variables contributing to PC1 | Time points A - D | Time points A – E | Time points B – D | Time points B – E | Time points C – D | Time points C - E |
| --- | --- | --- | --- | --- | --- | --- |
| IL-8 | 1.563791 | 2.828475 | 2.335840141 | 4.224903135 | 1.875723125 | 3.392675883 |
| IL-6 | 3.416386 | 4.513321 | 9.541231291 | 12.60473572 | 3.325756654 | 4.393592652 |
| IL-1β | 6.445444 | 6.265985 | 11.72976287 | 11.40317266 | 6.509991323 | 6.328734512 |
| PhciDAB | 4.045495 | 4.324019 | 3.409407839 | 3.644138631 | 2.998534349 | 3.204977337 |
| PhciDBB | 1.677595 | 1.353296606 | 1.684904954 | 1.35919376 | 2.173903231 | 1.753663137 |
Supplementary Table S3. Fold changes of immune variables contributing most to variation in PC1 of the PCA of time points A, B and C in the Liverpool Plains to time points D and E in the SHSWS population. Immune variables IL-8, IL-6, IL-1β, PhciDAB and PhiDBB contributed the most to PC1 and represented the innate response. These were significantly different between time points A, B and C to time points E and D: A vs D (t(103) = 4.041, p = <0.001), A vs E (t(103) = 5.248, p = <.0001), B vs D (t(103) = 4.642, p = <0.001), B vs E (t(103) = 5.683, p = <.0001), C vs D (t(103) = 4.181, p = <0.001) and C vs E (t(103) = 5.497, p = <.0001). For each target, fold changes were calculated using the average expression of the target in a time point divided by the average expression of the target in another time point e.g. ‘average expression IL-8 time point A/Average expression IL-8 time point D’

## Slide 9
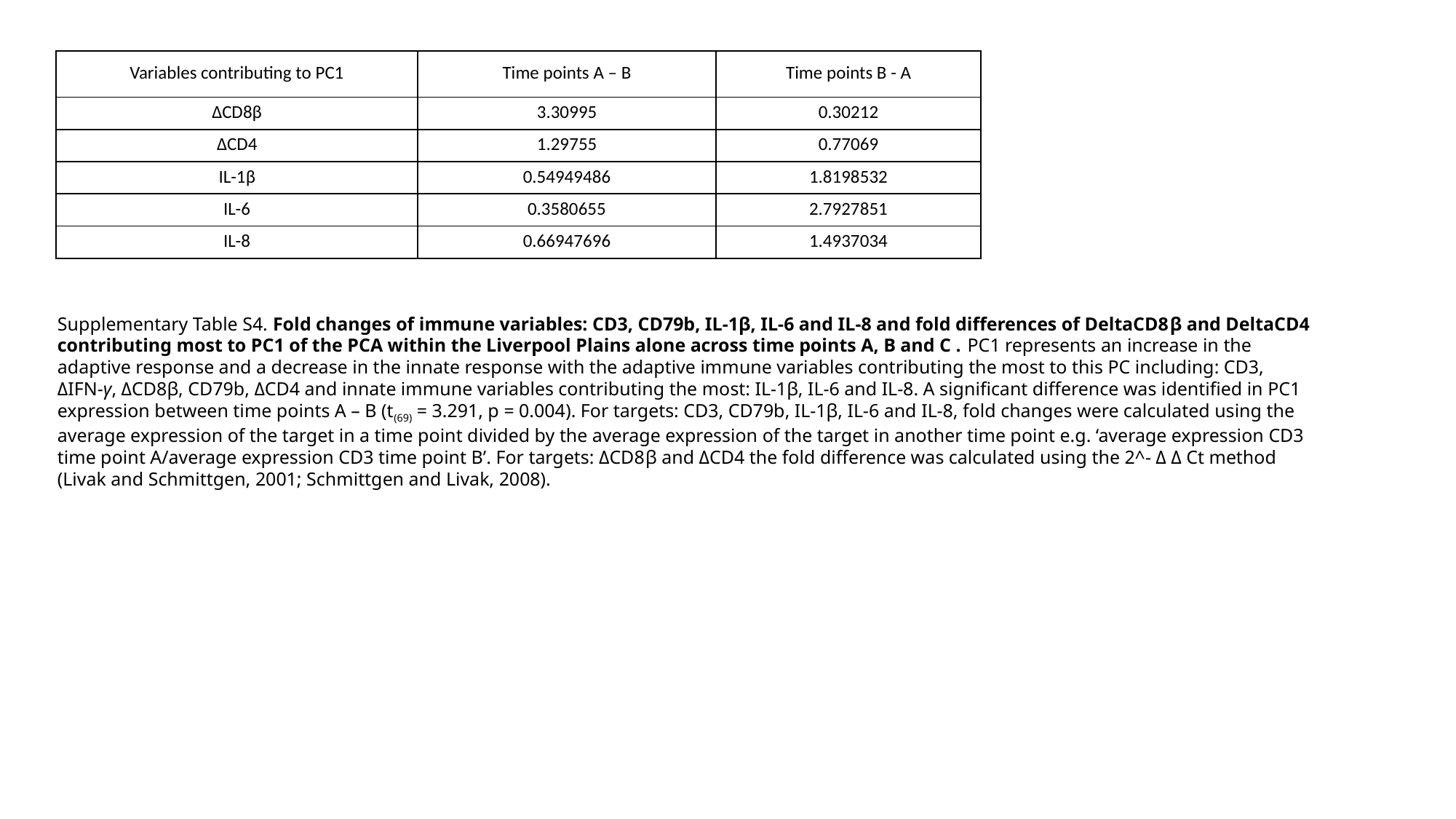

| Variables contributing to PC1 | Time points A – B | Time points B - A |
| --- | --- | --- |
| ΔCD8β | 3.30995 | 0.30212 |
| ΔCD4 | 1.29755 | 0.77069 |
| IL-1β | 0.54949486 | 1.8198532 |
| IL-6 | 0.3580655 | 2.7927851 |
| IL-8 | 0.66947696 | 1.4937034 |
Supplementary Table S4. Fold changes of immune variables: CD3, CD79b, IL-1β, IL-6 and IL-8 and fold differences of DeltaCD8β and DeltaCD4 contributing most to PC1 of the PCA within the Liverpool Plains alone across time points A, B and C . PC1 represents an increase in the adaptive response and a decrease in the innate response with the adaptive immune variables contributing the most to this PC including: CD3, ΔIFN-γ, ΔCD8β, CD79b, ΔCD4 and innate immune variables contributing the most: IL-1β, IL-6 and IL-8. A significant difference was identified in PC1 expression between time points A – B (t(69) = 3.291, p = 0.004). For targets: CD3, CD79b, IL-1β, IL-6 and IL-8, fold changes were calculated using the average expression of the target in a time point divided by the average expression of the target in another time point e.g. ‘average expression CD3 time point A/average expression CD3 time point B’. For targets: ΔCD8β and ΔCD4 the fold difference was calculated using the 2^- Δ Δ Ct method (Livak and Schmittgen, 2001; Schmittgen and Livak, 2008).
